# The Country Doctor and Aseptic Surgery*Read in the Section of Surgery of the Minnesota State Medical Society, June 16, 1898.

**Published:** 1898-11

**Authors:** P. A. Walling

**Affiliations:** Park Rapids, Minn.


### THE COUNTRY DOCTOR AND ASEPTIC SURGERY.*

BY P. A. WALLING, M.D., PARK RAPIDS, MINN.

That surgery has in the last decade made great strides toward perfection perhaps none realize more than the thinking country physician. That many diagnoses are made with clearness and precision which were only a few years ago obscure or doubtful is perhaps better appreciated by the city or hospital practitioner, but it is also realized fully by the alert country doctor.

Within the memory of my practice, all abdominal inflammations that were characterized by the usual sudden onset, pain, high temperature and tympanites were grouped together as one disease, and disposed of, generally by the undertaker, as "inflammation of the bowels." The abdominal cavity must under no circumstances be opened, and if by any accident the bowel was perforated, and the contents however small escaped, the case was considered as hopeless as though the heart had been punctured or the meninges exposed to the air.

To the city hospital surgeon all this has been changed; with modern knowledge of cleanliness, for which asepsis is but another term, the abdomen may be opened with as much impunity as a felon may be lanced; tumors and other morbid or septic material may be removed from the brain or spinal cord, and even the deeper recesses of the nerve centers may be exposed, and a recovery so far from being doubtful may be confidently expected.

Rules for asepsis, rules for disinfection, rules for this and rules for that are published without stint and almost without number, and it seems as though a wayfaring man though a fool could not err therein. This last expression is from the Holy Scriptures, and that is perhaps what makes it sound so new to most of you, and it makes me think of one more circumstance also from the same book, and that is what Haman remarked. After he had been honored far above everybody else and above what he deserved, he said: "All this availeth me nothing so long as I see Mordecai the Jew sitting at the king's gate."

I only change that to read: "All these rules and directions for asepsis and preparation avail me nothing when I am called to make a hurried operation that will not admit of a moment's delay and see a bedbug roosting on the head of the bed."

All these rules for asepsis, directions for cleaning the hands, washing the parts and getting ready for an operation are all right and as necessary to the success of an operation as can well be imagined, but I trow very few of those who insist on thorough asepsis realize, if they know, what the average frontier practitioner has to contend with.

*Read in the Section of Surgery of the Minnesota State Medical Society, June 16, 1898.

It is the object of this very imperfect paper to note some of the points where we succeed without any preparation and where a typical city man would fail.

I will try in the first place to draw a pen picture of an average frontier country home: The house is usually built of logs without a foundation and covered with boards, shingles or shakes. The walls are unplastered and usually covered with bedding or newspapers and are absolutely unclean, generally filled with dust and garrisoned by cimex lectularius or pulex irritans, which Conkey defines as two too well-known insects. Floors are of loose boards and above all are generally open, while the lower floor is usually lined with paper to keep out the cold, and this paper is often saturated with water from a dirty mop or, worse, with the excretions of children who are often untaught in the ways of civilized decency. Personally the people correspond with their surroundings. Their washing is done carelessly, often without boiling; bodies are unbathed except perhaps the male members bathe during the log driving season, the same as a pioneer Western man dies with his boots on; dogs and cats lie under or upon the bed, and fleas, et cetera, are a seemingly large part of the population.

Absolute ignorance of the simplest sanitary principles and seeming inability to comprehend what cleanliness is render requests for clean water and clean towels inert. In asking for the foregoing articles I have had a wash dish brought to me with a rim of dirt at the water line, two or three rags drawn in at the bottom and a towel at the sight of which a printer would die of envy. These people are poor; they are not really to blame for this condition, for they are driven to seek the barest necessities of life in the Northwestern wilds, and were long since obliged to be satisfied with what in its simplest form would support life.

This class of people have many accidents and raise many children; they are frequently ten to thirty miles from any place where cleanliness is at all observed, and if not so it is often an impossibility to have them removed at all.

I will give you one instance of surgery under almost the identical circumstances enumerated above.

A young man was driving a log measuring about 500 feet through the woods one afternoon, when he being on the load, it tipped, throwing him to the ground and pinning one leg between the log and the binding pole on the hard frozen ground. He was picked up and taken to the house, where I found him a little after dark, lying on the floor, with a compound fracture of both bones just above the ankle joint.

A small kerosene lamp afforded all the light, and an examination revealed a very serious condition under any circumstances and made infinitely more so by the surroundings.

Should I amputate? seemed the only question, and had I been ready I believe that leg would have gone to the boneyard, before morning. I was not prepared without going eight miles for help and more instruments, and so decided to try to save it. I asked for cloth for bandage and was supplied with those which your dry goods men here in the city would hate to own once belonged to them.

I had plenty of carbolic acid and bichloride of mercury and made appropriate solutions of each and drew that leg into position and dressed it in a fracture box and bandages with a liberal supply of both antiseptics, and left him late at night comparatively easy.

Do not ask me if any pus accumulated in this wound or I shall be obliged to lower my dignity and descend to slang. I visited that case every other day for about four weeks, not trusting any one else to dress it, and often found puddles of pus an inch in depth alongside of the wound. At each visit I dressed and cleaned it up, taking my own bandages after the first visit, and had the satisfaction of saving the foot, and though it is united in rather bad shape, he walks about as well as ever. With the five dollars and the chicken I received for this work, I made a meal and with the balance bought more antiseptics for the next case.

In such cases as these, so far removed and under such circumstances, cleanliness is not to be considered; for if you touch anything you only raise a dust and bring it, if possible, into more septic conditions. Do not clean the dirt from under your finger nails, but cover it down with a little oil; do not wash your hands, for they are no doubt dirty enough now; do not scrub down the walls or the floor, for if you let the denizens of this territory alone they will come nearer letting you alone.

When we studied medicine Dr. Julius F. Miner told us the first surgeons were not those who could make a successful job when they had all the modern applicances and surroundings, but the man who, when far away from patent splints, could with his jackknife and a fence rail make the apparatus that would properly secure a fracture.

A man to practice surgery in the country may. if he likes, learn all there is of modern methods and all the details of asepsis; but if he thinks he must have all these things at hand for every case he will oftener find he is without resources than otherwise. I do not wish to be understood as ridiculing modern asepsis or modern methods. On the other hand, I consider the advance of the last ten years as wonderful beyond comparison, and the good it has accomplished and lives it has saved will never be known; but what I wish to say to the country practitioner is, because you can not have and use all the modern improvements do not be discouraged, but do the best possible under the circumstances, and if you succeed, well; if you do not, the fau t lies not with you.—*Northwestern Lancet.*

